
JOURNAL INFORMATION
==============================
NLM Title Abbreviation: Medizinrecht
Journal ID: Medizinrecht
NLM Journal ID: 8309354

Medizinrecht

ISSN: 0723-8886
EISSN: 1433-8629

ARTICLE INFORMATION
==============================
PMCID: PMC8918893
PMID: 35310872
DOI: 10.1007/s00350-022-6134-9
Article ID: 6134
Article version: 1
Subjects: Aufsätze

Benachteiligungsrisiken von Menschen mit Behinderung in der Triage – effektive Abwehr aus Karlsruhe?

Zugleich Besprechung von zu BVerfG, Beschl. v. 16.12.2021 – 1 BvR 1541/20

Taupitz Jochen
grid.506582.c 0000 0001 2228 994X Institut für Deutsches, Europäisches und Internationales Medizinrecht, Gesundheitsrecht und Bioethik, Universitäten Heidelberg und Mannheim, Schloss, 68131 Mannheim, Deutschland
Electronic publication date: 2022 Mar 14
Print publication date: 2022
Volume: 40
Issue: 3
First page: 181
Last page: 185
Copyright: © Der/die Autor(en) 2022
License: Open Access Dieser Artikel wird unter der Creative Commons Namensnennung 4.0 International Lizenz veröffentlicht, welche die Nutzung, Vervielfältigung, Bearbeitung, Verbreitung und Wiedergabe in jeglichem Medium und Format erlaubt, sofern Sie den/die ursprünglichen Autor(en) und die Quelle ordnungsgemäß nennen, einen Link zur Creative Commons Lizenz beifügen und angeben, ob Änderungen vorgenommen wurden.
Die in diesem Artikel enthaltenen Bilder und sonstiges Drittmaterial unterliegen ebenfalls der genannten Creative Commons Lizenz, sofern sich aus der Abbildungslegende nichts anderes ergibt. Sofern das betreffende Material nicht unter der genannten Creative Commons Lizenz steht und die betreffende Handlung nicht nach gesetzlichen Vorschriften erlaubt ist, ist für die oben aufgeführten Weiterverwendungen des Materials die Einwilligung des jeweiligen Rechteinhabers einzuholen.
Weitere Details zur Lizenz entnehmen Sie bitte der Lizenzinformation auf http://creativecommons.org/licenses/by/4.0/deed.de.
License URL: https://creativecommons.org/licenses/by/4.0/

issue-copyright-statement: © Springer-Verlag GmbH Deutschland, ein Teil von Springer Nature 2022

==============================

**Jochen Taupitz**

*Besteht für Menschen mit Behinderung ein reales Risiko, bei der Entscheidung über die Zuteilung von überlebenswichtigen intensivmedizinischen Ressourcen jedenfalls dann benachteiligt zu werden, wenn diese pandemiebedingt knapp sind? Und wenn ja: Wie kann diesem Risiko wirksam begegnet werden? Mit beiden Fragen befasst sich die Entscheidung des BVerfG vom 16. 12. 2021 – allerdings in sehr unterschiedlicher Intensität.*

### I. Einleitung

Die Frage, nach welchen Kriterien knappe intensivmedizinische Ressourcen wie z.B. Beatmungsgeräte in der aktuellen Corona-Pandemie zu verteilen und damit zuzuteilen sind, wird spätestens seit den bedrückenden Berichten über Ereignisse etwa in Bologna 2020 international intensiv diskutiert[1]. Dabei wurde auf die spezielle Situation von Menschen mit Behinderung zumeist eher am Rande eingegangen. Das hat sich spätestens mit der Verfassungsbeschwerde von Menschen mit Behinderung v. 27.6.2020 geändert[2]. Während das BVerfG noch mit Beschluss v. 16.7.2020 einen Antrag auf Erlass einer einstweiligen Anordnung zurückgewiesen hatte, weil zu diesem Zeitpunkt nicht konkret absehbar gewesen sei, dass die Plätze für eine intensivmedizinische Behandlung in den (deutschen) Krankenhäusern nicht ausreichen würden, um notwendige Maßnahmen für alle Behandlungsbedürftigen zu ergreifen[3], hat das Gericht jetzt in seiner (in diesem Heft abgedruckten) Entscheidung in der Hauptsache ein Risiko der Benachteiligung wegen einer Behinderung bei der Zuteilung knapper, überlebenswichtiger intensivmedizinischer Ressourcen bejaht.

### II. Aus Art. 3 Abs. 3 S. 2 GG folgende Handlungspflichten des Gesetzgebers

Der Ausgangspunkt der Entscheidung des BVerfG ist wenig überraschend: Aus Art. 3 Abs. 3 S. 2 GG ergibt sich für den Staat nicht nur das Verbot unmittelbarer und mittelbarer Diskriminierungen wegen Behinderung, sondern auch ein Auftrag, Menschen wirksam vor Benachteiligung wegen ihrer Behinderung durch Dritte zu schützen (Rdnr. 96)[4]. Und dieser Schutzauftrag kann sich in bestimmten Konstellationen ausgeprägter Schutzbedürftigkeit zu einer konkreten Schutzpflicht verdichten. Dazu gehören neben gezielten, als Angriff auf die Menschenwürde zu wertende Ausgrenzungen von Personen wegen einer Behinderung auch eine mit der Benachteiligung wegen Behinderung einhergehende Gefahr für hochrangige grundrechtlich geschützte Rechtsgüter wie das Leben sowie Situationen struktureller Ungleichheit (Rdnr. 97). Das alles wird vom Gericht akribisch mit Zitaten belegt. Nicht angesprochen – da offenbar nicht entscheidungserheblich – wird dagegen, inwieweit sich ein Schutzauftrag auch unmittelbar aus Art. 2 Abs. 2 S. 1 GG ableiten ließe[5].

### III. Umstrittene Fragen – Teilantworten des Gerichts

1. Heftig umstrittene, vom Verfassungsgericht bisher nicht geklärte zentrale Fragen der Verteilung und damit Zuteilung knapper, überlebenswichtiger intensivmedizinischer Ressourcen[6] werden vom Gericht dagegen eher nonchalant, fast nebenbei beantwortet. Dies widerspricht der Tradition deutscher Gerichte und auch des BVerfG, sich mit prominent vertretenen Gegenauffassungen argumentativ auseinanderzusetzen. Dazu gehört vorliegend insbesondere die Frage, ob der Staat (abgesehen vom Bereich des verfassungsrechtlich insbesondere im Hinblick auf Art. 3 Abs. 3 GG *nicht* Zulässigen) überhaupt abstrakt bindende Vorgaben für die Allokation knapper Ressourcen machen und dabei das von den medizinischen Fachgesellschaften zumeist in das Zentrum ihrer Empfehlungen gestellte Kriterium der klinischen Erfolgsaussicht[7] vorgeben darf. Dies wurde insbesondere vom Deutschen Ethikrat vehement bestritten[8]:

„Diese [verfassungsrechtlichen] Vorgaben widerstreiten einem rein utilitaristischen Modus des Abwägens im Sinne

Prof. Dr. iur. Jochen Taupitz,
Geschäftsführender Direktor des Instituts für Deutsches, Europäisches und Internationales Medizinrecht, Gesundheitsrecht und Bioethik
der Universitäten Heidelberg und Mannheim,
Schloss, 68131 Mannheim, Deutschland

1) S. etwa *Taupitz*, MedR 2020, 440 ff.; ferner die Beiträge in *Woesler/Sass* (Hrsg.), Medizin und Ethik in Zeiten von Corona, 2020.
2) Die Antragsschrift ist abrufbar unter https://www.menschenundrechte.de/uploads/media/Verfassungsbeschwerde_COVID_19_Triage_Az_1_BvR_1541_20_HP.pdf, Zugriff am 7.1.2022.
3) NVwZ 2020, 1353; dazu *Brade/Müller*, NVwZ 2020, 1792 ff.
4) Die Angaben der Randnummern beziehen sich auf die Entscheidung des BVerfG.
5) Dazu *Brade/Müller*, NVwZ 2020, 1792, 1794 f., 1797. Auf die „Schutzpflichten für das Leben und die Gesundheit" (übrigens auch „der anderen Patientinnen und Patienten" neben den Menschen mit Behinderung) weist das Gericht lediglich beiläufig Rdnr. 127 hin, darüber hinaus auf die „Bedeutung des Art. 2 Abs. 2 S. 1 GG" in Rdnr. 130.
6) Das Gericht betont selbst (Rdnr. 85), dass die Verfassungsbeschwerde „zentrale verfassungsrechtliche Fragen" aufwerfe.
7) S. etwa die DIVI-Empfehlungen „Entscheidungen über die Zuteilung intensivmedizinischer Ressourcen im Kontext der COVID-19-Pandemie – Klinisch-ethische Empfehlungen", Stand: 14.12.2021, abrufbar unter https://www.awmf.org/leitlinien/detail/ll/040-013.html, Zugriff am 7.1.2022; auf die Empfehlungen (in einer früheren Fassung) sowie auf DIVI-Pressemitteilungen dazu geht das BVerfG in seiner Entscheidung mehrfach ein (Rdnrn. 114–116; 119, 120, 128); Überblick über weitere in- und ausländische Stellungnahmen von Fachgesellschaften bei *Taupitz*, MedR 2020, 440, 443; s. ferner die „Orientierungshilfe der Bundesärztekammer zur Allokation medizinischer Ressourcen am Beispiel der SARS-CoV-2-Pandemie im Falle eines Kapazitätsmangels", DÄBl. 2020, A 1084 ff.
8) Deutscher Ethikrat, Solidarität und Verantwortung in der Corona-Krise, S. 3 f., abrufbar unter https://www.ethikrat.org/fileadmin/Publikationen/Ad-hoc-Empfehlungen/deutsch/ad-hoc-empfehlung-corona-krise.pdf, Zugriff am 7.1.2022.

einer bloßen Maximierung von Menschenleben […]. Der Staat darf menschliches Leben nicht bewerten, und deshalb auch nicht vorschreiben, welches Leben in einer Konfliktsituation vorrangig zu retten ist. Selbst in Ausnahmezeiten eines flächendeckenden und katastrophalen Notstands hat er nicht nur die Pflicht, möglichst viele Menschenleben zu retten, sondern auch und vor allem die Grundlagen der Rechtsordnung zu garantieren. […] Das bedeutet nicht, dass keine handlungsleitenden Vorgaben konzipierbar wären. Aus dem Verbot einer eigenen staatlichen Bewertung folgt nicht, dass entsprechende Entscheidungen nicht akzeptiert werden können. Es ist daher sinnvoll, unterschiedliche Ebenen normativer Konkretisierung zu verbinden. Das verweist auf die Funktion zum Beispiel der Fachgesellschaften, die im Rahmen der vorgenannten Grundvorgaben wichtige Orientierungshilfen geben können und sollten, welche inhaltlich über das hinausgehen, was staatlicherseits zulässig wäre. Die damit hervorgehobene Primärverantwortung der Medizin für einzelne Entscheidungen und deren Umsetzung folgt aus den erwähnten Grenzen des staatlich Normierbaren, die dem Verfassungsrecht geschuldet sind. Doch sollte diese Verantwortung keinesfalls nur ‚am Krankenbett', also im Rahmen der individuellen Arzt-Patienten-Beziehung, wahrgenommen werden. Schon aus Gründen der Gleichbehandlung, aber auch um der allgemeinen Akzeptanz willen bedarf es vielmehr weithin einheitlicher Handlungsmaximen für den klinischen Ernstfall. In der Reaktion auf die aktuelle Krise sind […] erste Empfehlungen medizinischer Fachgesellschaften erschienen."

Die damit vom Ethikrat verneinte *staatliche* Zuständigkeit würde nicht demokratisch legitimierten Institutionen – ohne realistische Aussicht auf (vorbeugenden) Rechtsschutz[9] – die Primärverantwortung zuschieben. Die vom Ethikrat postulierte gesetzgeberische Unzuständigkeit gipfelte nach Auffassung des Ethikrates zugleich darin, dass sich ein Mediziner, der eine bestimmte[10] „Gewissensentscheidung trifft, die ethisch begründbar ist und transparenten – etwa von medizinischen Fachgesellschaften aufgestellten – Kriterien folgt", dem Risiko der Verurteilung wegen einer rechtswidrigen Straftat aussetzt und allenfalls mit „entschuldigender Nachsicht der Rechtsordnung rechnen" darf; denn der Staat habe auch in Katastrophenzeiten „die Fundamente der Rechtsordnung zu sichern"[11]. Dass von Seiten der Ärzte eine „unerträgliche Rechtsunsicherheit" beklagt wird, ist vor diesem Hintergrund mehr als verständlich.

Von all dem liest man in der Entscheidung des BVerfG nichts. Die Stellungnahme des Ethikrates wird vom Gericht lediglich zu Beginn der Entscheidung (Rdnr. 7) erwähnt: „Hinweise des Deutschen Ethikrates (Solidarität und Verantwortung in der Corona-Krise – Ad-hoc-Empfehlung, 27. 3. 2020)" seien in der Praxis neben notärztlichen ‚Leitplanken' der Bundesvereinigung der Arbeitsgemeinschaften der Notärzte Deutschlands (BAND) e. V. „von Bedeutung". Und die von der DIVI im Verfahren beklagte „unerträgliche" Rechtsunsicherheit für die Ärzte, „welche Kriterien im Fall einer Pandemie bei der Verteilung knapper medizinischer Ressourcen maßgeblich sein sollen", wird vom Gericht (Rdnr. 120) lediglich als Argument benutzt, um das u. a. daraus resultierende Risiko einer Benachteiligung von Menschen mit Behinderung zu begründen. Stattdessen wird die gesetzgeberische Handlungspflicht ohne jede Problematisierung staatlicher Handlungs*befugnisse* aus Art. 3 Abs. 3 S. 3 GG abgeleitet. Und selbst hinsichtlich der gesetzgeberischen Handlungspflicht wäre es auch im Hinblick auf andere von medizinischen Zuteilungsentscheidungen Betroffene (über den Kreis der Menschen mit Behinderung hinaus) hilfreich gewesen, wenn das Gericht eine Begründung auch über die Wesentlichkeitslehre auf der Grundlage des Gesetzesvorbehalts geliefert hätte[12]. Mit dieser Begründung hätte z. B. auch eine Antwort auf die bereits häufig[13] gestellte Frage gegeben werden können, was denn der Vorteil einer gesetzgeberischen Entscheidung im Vergleich zu Empfehlungen der medizinischen Fachgesellschaften sein soll.

Bezüglich der zentralen Aussage, dass das der medizinischen Praxis zugrunde liegende Kriterium der klinischen Erfolgsaussicht (und damit das Ziel einer Maximierung der Überlebendenzahl) nicht nur verfassungsrechtlich zulässig, sondern sogar „verfassungsrechtlich unbedenklich" ist (Rdnr. 118), also auch vom Gesetzgeber vorgegeben werden darf (Rdnr. 128[14]), begnügt sich das Gericht mit zwei mageren Sätzen: „Die Aussicht, die akute Erkrankung zu überleben, ist ein als solches zulässiges Auswahlkriterium für die Verteilung knapper Behandlungsressourcen. Dieses Kriterium stellt nicht auf eine Bewertung menschlichen Lebens ab, sondern allein auf die Erfolgsaussichten der nach der aktuellen Erkrankung angezeigten Intensivtherapie" (Rdnr. 116). Gerade dies (und insbesondere die Aufnahme dieses Kriteriums in ein Gesetz) wurde und wird von Teilen der Literatur[15] und auch vom Deutschen Ethikrat (s. oben) anders gesehen. So wird etwa in der Literatur kritisiert, dass das menschliche Leben seines subjektiven, einzigartigen Charakters beraubt werde, wenn ein „Aussortierter" nur noch Zählwert habe; Menschenleben dürften nicht gegeneinander abgewogen werden[16]. Einziger Aussonderungsgrund könne sein, dass die Behandlung aussichtslos

9) Dazu Rdnr. 84 der Entscheidung des BVerfG: „Hier ist nicht erkennbar, wie in zumutbarer Weise eine gerichtliche Entscheidung darüber zu erlangen wäre, in der akuten Notsituation einer im Laufe der Coronavirus-Pandemie eventuell notwendigen Triage in der Intensivmedizin nicht aufgrund einer Behinderung zurückgestellt … zu werden. Unabhängig von der Frage, ob derartige Behandlungsansprüche überhaupt einklagbar wären, kämen gerichtliche Eilentscheidungen im akuten Fall zu spät. Die nachträgliche Überprüfung einer ärztlichen Entscheidung ist hier nicht zielführend. Auch vorbeugender Rechtsschutz dagegen, im Fall einer intensivmedizinischen Knappheit aufgrund einer Behinderung benachteiligt zu werden, existiert realistisch nicht."

10) Nämlich (aus dem Blickwinkel der klinischen Erfolgsaussicht) das Beenden der Beatmung eines Patienten und stattdessen Beatmung eines anderen (später hinzukommenden) Patienten mit besserer Überlebenschance bei nur einem verfügbaren Beatmungsgerät (sog. ex-post-Konstellation). S. zu den verschiedenen Triage-Konstellationen noch unten IV. 1.

11) Deutscher Ethikrat, Solidarität und Verantwortung in der Corona-Krise, S. 4, abrufbar unter https://www.ethikrat.org/fileadmin/Publikationen/Ad-hoc-Empfehlungen/deutsch/ad-hoc-empfehlung-corona-krise.pdf, Zugriff am 7. 1. 2022. Kritisch – sowie ex-ante und ex-post Konstellation im Grundsatz gleichbehandelnd – *Gaede* et al., medstra 2020 129, 134 ff.; *Hoven/Hahn*, JA 2020, 482, 483 f.; *Hoven*, JZ 2020, 449, 453 f.; *Jäger/Gründel*, ZIS 2020, 151, 156 ff.; *Kersten/Rixen*, Der Verfassungsstaat in der Corona-Krise, 2. Aufl. 2021, VI. 2. Triage; *Taupitz*, MedR 2020, 440, 447. – Neuere Übersicht zur Diskussion bei *Spickhoff*, NJW 2021, 1713, 1714 f.

12) Zur Notwendigkeit einer gesetzlichen Regelung *Kersten/Rixen*, Der Verfassungsstaat in der Corona-Krise, 2. Aufl. 2021, VI. 2. Triage; *Bockholdt*, in: *Schlegel/Meßling/Bockholdt*, COVID-19 – Corona-Gesetzgebung – Gesundheit und Soziales, 2020, § 9, Rdnrn. 138 f.; *Taupitz*, MedR 2020, 440, 441 f.; zur Diskussion um das Für und Wider einer gesetzlichen Regelung s. näher *Huster/Kingreen*, Handbuch Infektionsschutzrecht, 2021, Kap. 8, Rdnrn. 41, 52 ff.

13) S. schon vor der Entscheidung des BVerfG die bei *Ehlers* et al. (MedR 2021, 416, 417) wiedergegebenen Stimmen.

14) Denn es heißt dort: „ein Kriterium, das den inhaltlichen Anforderungen der Verfassung genügt, kann vom Gesetzgeber vorgegeben werden"; genau dies trifft nach Aussage des Gerichts für das „verfassungsrechtlich unbedenklich[e]" Kriterium der klinischen Erfolgsaussicht zu.

15) S. aus neuester Zeit etwa *Merkel/Augsberg*, JZ 2020, 704 ff.; *Engländer/Zimmermann*, NJW 2020, 1398, 1401 f.; *Sternberg-Lieben*, MedR 2020, 627, 632; *Rönnau/Wegner*, Jus 2020, 403, 405.

16) S. die Nachweise zu entsprechenden Stimmen bei *Taupitz*, MedR 2020, 440, 445; ferner die Nachweise in der folgenden Fußnote.

ist[17]. Angesichts dessen hätte man eine Auseinandersetzung mit der Gegenauffassung durchaus erwarten können, auch wenn dem Ergebnis des Gerichts uneingeschränkt zuzustimmen ist[18].

2. Im Ergebnis zutreffend[19], wenn auch wiederum ohne jede Auseinandersetzung mit Gegenstimmen[20], sowie in der Tragweite offen sind die Ausführungen des Gerichts zur Unzulässigkeit des Abstellens auf die zu erwartende Überlebensdauer jenseits des Überlebens der aktuellen Erkrankung: „Würde hingegen auf eine längerfristig erwartbare Überlebensdauer abgestellt, würden Menschen, die aufgrund von Behinderungen tatsächlich oder vermeintlich eine kürzere Lebenserwartung haben, regelmäßig nicht oder nachrangig behandelt, zumal die stereotype Wahrnehmung von Behinderungen zu vorschnellen Schlüssen auf eine kürzere Lebensdauer verleiten kann. Dann wäre die weitere Lebensperspektive ausschlaggebend, nicht aber die Aussicht, die aktuelle Erkrankung zu überleben. Es ginge dann gerade nicht um das Überleben der akuten Erkrankung, sondern um die Maximierung von Lebenszeit“ (Rdnr. 117). Ob diese auf Menschen mit Behinderung zielende Aussage – wegen des von den Beschwerdeführern gestellten Antrags[21] nicht entscheidungserheblich – zugleich eine generelle Absage an Auswahlentscheidungen abhängig vom Lebensalter von Patienten bedeutet (wobei das Alter immerhin nicht im Katalog des Art. 3 Abs. 3 GG als unzulässiges Kriterium aufgeführt ist), wird nicht angesprochen[22]. Wiederum lediglich en passant und nur bezogen auf Menschen mit Behinderung erteilt das Gericht – im Gegensatz etwa zu WHO[23] – eine Absage an das Abstellen auf eine Maximierung „qualitätsbereinigter Lebensjahre“ (QALYS – „quality adjusted life years saved“) (Rdnr. 118)[24].

### IV. Reaktionen des Gesetzgebers?

1. Spannend bleibt, welche Maßnahmen der Gesetzgeber nun tatsächlich ergreifen wird – und ob die letztlich getroffene Regelung einer dagegen wohl zu erwartenden weiteren Verfassungsbeschwerde standhalten wird. Denn dass es dem Gesetzgeber gelingen wird, „*jede* Benachteiligung wegen einer Behinderung bei der Verteilung pandemiebedingt knapper intensivmedizinischer Behandlungsressourcen hinreichend wirksam“ zu verhindern (so die Forderung des Gerichts Rdnr. 130[25]), ist kaum zu erwarten. Immerhin leitet das Gericht das Risiko, wegen einer Behinderung bei der Verteilung knapper intensivmedizinischer Ressourcen benachteiligt zu werden, aus einer „Gesamtschau verschiedener, teils ineinandergreifender Umstände“ ab (Rdnr. 126; zur „Gesamtschau“ auch Rdnrn. 110, 121). Genannt werden (Rdnrn. 111, 113, 120) u. a. „subjektive Momente […] in der komplexen Entscheidung über eine intensivmedizinische Therapie […], die Diskriminierungsrisiken beinhalteten“, „dass die Lebenssituation und -qualität von Menschen mit Behinderungen oft sachlich falsch beurteilt“ werde, dass das „in Krankenhäusern tätige[…] Personal […] meist unter hohem zeitlichen und ökonomischen Druck [stehe] und […] im Umgang mit Menschen mit spezifischen Behinderungen in der Regel nicht geschult“ sei, dass das Diskriminierungsrisiko „aus mangelndem Fachwissen und einer unzureichenden Sensibilisierung des medizinischen und pflegenden Gesundheitspersonals für behinderungsspezifische Besonderheiten“ resultiere, „eine unbewusste Stereotypisierung von behinderten Menschen diese bei medizinischen Entscheidungen benachteilige“ und der „medizinische Blick auf Behinderung […] häufig defizitorientiert […] [sei], was in der Ausbildung bislang kaum bearbeitet werde“. Ähnlich wird im Hinblick auf im Grundsatz ausdrücklich als zulässig bezeichnete Kriterien (Komorbiditäten, Rdnr. 118) bzw. Hilfsmittel (Verwendung einer Skala, Rdnr. 118), befürchtet, dass sie „pauschal“, „stereotyp“ oder „vorschnell“ einer Zuteilungsentscheidung zugrunde gelegt würden (Rdnr. 118) bzw. dass eine Benachteiligung von Menschen mit Behinderung in der Praxis nicht „ausgeschlossen“ werden könne (zwei Mal Rdnr. 118).

Diesen Risiken wird der Gesetzgeber kaum durch die vom Gericht ausdrücklich für möglich gehaltene „Regelung von Kriterien, nach denen zu entscheiden ist, wie knappe Ressourcen zur Lebensrettung verteilt werden“ (Rdnr. 128), hinreichend begegnen können. Denn wie auch immer sie ausgestaltet werden, kann ihre fehlerhafte (benachteiligende) Anwendung im täglichen praktischen Vollzug doch ganz sicher „nicht ausgeschlossen“ werden. Eine weitere Verfassungsbeschwerde ist damit vorprogrammiert.

Zudem bleibt in der Entscheidung des Gerichts völlig offen, welche Kriterien und in welcher Reihenfolge abgesehen von der „verfassungsrechtlich unbedenklichen“ klinischen Erfolgsaussicht im Sinne des Überlebens der aktuellen Erkrankung (Rdnrn. 116, 118)[26] und abgesehen von *unzulässigen* Kriterien (Benachteiligung wegen einer Behinderung, Art. 3 Abs. 3 S. 2 GG, [Rdnrn. 88 ff.] bzw. längerfristig erwartbare Überlebensdauer, QALYS, [Rdnrn. 117, 118]) vom Gesetzgeber vorgegeben werden dürfen. Denn das Gericht begnügt sich insoweit mit der Aussage: „Dass aufgrund der Achtung vor der Unantastbarkeit der Menschenwürde Leben nicht gegen Leben abgewogen werden darf […], steht einer Regelung von Kriterien, nach denen zu entscheiden ist, wie knappe Ressourcen zur Lebensrettung verteilt werden, nicht von vornherein entgegen; ein Kriterium, das den inhaltlichen Anforderungen der Verfassung genügt, kann vom Gesetzgeber vorgegeben werden“ (Rdnr. 128). Damit ist insbesondere (weiter) offen, in welchem Verhältnis z. B. das neben der Erfolgsaussicht in § 12 Abs. 3 S. 1 TPG genannte und allgemein als zulässig angesehene Kriterium der Dringlichkeit[27] zur Erfolgsaussicht stehen darf[28] – eine nicht nur in der aktuellen Diskussion sehr umstrittene Frage. Es ist kaum zu erwarten, dass der Gesetzgeber mit einer Regelung aus Anlass der vorliegenden Entscheidung zugleich das Fass einer Neuregelung des TPG aufmachen wird.

Aus einem weiteren Grund dürfte die Neigung des Gesetzgebers nicht sehr groß sein, sich durch die Vorgabe bestimm-

17) *Lindner*, MedR 2020, 723, 727 sowie *Engländer/Zimmermann*, NJW 2020, 1398, 1402: Zurückstellen eines Patienten allenfalls dann, wenn er nach medizinischer Einschätzung „keine oder nur sehr geringe Aussichten“ hat, trotz der Zuteilung des Rettungsmittels zu überleben; *Sternberg-Lieben*, MedR 2020, 627, 631 f.: Nur bei „Aussichtslosigkeit der Heilbehandlung (kein Mindestnutzen)“.

18) *Taupitz*, MedR 2020, 440, 445 f.; s. auch den entsprechenden Gesetzesvorschlag von *Kersten/Rixen*, unten IV. 5. – Das bedeutet freilich nicht, dass das Kriterium der klinischen Erfolgsaussicht vom Gesetzgeber als bindend vorgegeben werden *muss* bzw. als *einziges* Kriterium rechtlich vorgegeben werden kann, s. dazu noch unten IV. 1. – Abgesehen davon ist die Zulässigkeit einer gesetzgeberischen Lösung nicht mit der Frage zu verwechseln, ob die klinische Erfolgsaussicht bereits de lege lata als Vorrangkriterium fungiert, s. sehr deutlich *Sowada*, NStZ 2020, 452, 455.

19) *Taupitz*, MedR 2020, 440, 448.

20) Etwa *Hoven*, JZ 2020, 449, 451 f.; zur Diskussion s. *Sternberg-Lieben*, MedR 2020, 627, 631 ff.

21) Oben Fn. 2.

22) S. aber BVerfGE 115, 118, 158: „Menschliches Leben und menschliche Würde genießen ohne Rücksicht auf die Dauer der physischen Existenz des einzelnen Menschen gleichen verfassungsrechtlichen Schutz“.

23) WHO, Guidance for Managing Ethical Issues in Infectious Disease Outbreaks, 2016, S. 21, abrufbar unter https://apps.who.int/iris/bitstream/handle/10665/250580/9789241549837-eng.pdf;jsessionid=2FB63D1A7179EB93BFCA087FEEB7EE2F?sequence=1, Zugriff am 7. 1. 2022.

24) Dazu *Taupitz*, MedR 2020, 440, 448.

25) Hervorhebung vom Verfasser.

26) Der Regelungsvorschlag von *Kersten/Rixen* (unten IV. 5) beschränkt sich auf die Anordnung der diskriminierungsfreien Anwendung des Kriteriums der klinischen Erfolgsaussicht.

27) Dazu *Taupitz*, MedR 2020, 440, 444.

28) S. zur Diskussion bezogen auf die Triage hier nur *Ehlers* et al., MedR 2021, 416, 419 ff.; *Gerson*, in: *Esser/Tsambikakis*, Pandemiestrafrecht, 2020, § 3, Rdnrn. 22–27, 71–74, jew. m. w. N.

ter Verteilungskriterien „die Finger zu verbrennen"; er wird sich deshalb wahrscheinlich auf andere Maßnahmen (unten IV. 2.) beschränken. Das BVerfG erwähnt im Rahmen seiner Risikoanalyse nämlich mit keinem Wort, in welchen der in der Literatur und vom Deutschen Ethikrat heftig diskutierten Triage-Situationen überhaupt das Risiko einer Benachteiligung von Menschen mit Behinderung besteht, so dass *diesem* Risiko von Seiten des Gesetzgebers wirksam entgegenzuwirken ist: Ist es die Situation der sog. ex-ante-Triage[29], in der zwei Patienten, die auf überlebenswichtige intensivmedizinische Maßnahmen angewiesen sind, *gleichzeitig* in die Klinik (Notaufnahme? Intensivstation?) eingeliefert werden und sich lediglich hinsichtlich der klinischen Erfolgsaussicht unterscheiden? Geht es um die Situation eines Patienten, der bereits beatmet wird, dem aber ein *später* hinzukommender Patient mit (etwas? deutlich?) besserer klinischer Erfolgsaussicht vorgezogen wird, indem die künstliche Beatmung des ersten Patienten zugunsten des zweiten Patienten beendet wird („ex-post-Triage")? Gerade diese Situation steht ja ganz im Zentrum der juristischen Diskussion. Oder steht schließlich eine Situation in Frage, in der einem Patienten mit relativ schlechter Prognose das einzig noch verfügbare Beatmungsgerät vorenthalten wird, weil ein Patient mit besserer Prognose (alsbald? in naher Zukunft?) erwartet wird („präventive Triage")? Da die ex-ante-Triage in der Praxis sehr selten sein dürfte und die präventive Triage von der Literatur, soweit ersichtlich, einhellig abgelehnt wird, müsste sich der Gesetzgeber, wenn er denn tatsächlich praktisch relevante Verteilungskriterien für die Triage vorgeben würde, jedenfalls auch mit der ex-post-Triage befassen[30]. Es ist zu prognostizieren, dass er dieses heikle Thema nicht anfassen wird.

2. Der Gesetzgeber wird sich wohl eher auf die vom Gericht für möglich gehaltenen „Vorgaben zum Verfahren", ein „Mehraugen-Prinzip bei Auswahlentscheidungen", „Vorgaben zur Dokumentation" und/oder „Regelungen zur Unterstützung vor Ort" (Rdnr. 128) fokussieren. Da insoweit zentral die ärztliche Berufsausübung betroffen sein wird – denn es sind vor allem Ärzte, die über die Zuteilung von intensivmedizinischen Ressourcen entscheiden –, ist allerdings zu bedenken, dass insoweit keine Bundeskompetenz besteht. „Der" zur Regelung aufgerufene „Gesetzgeber" bedeutet deshalb wohl „die Landesgesetzgeber", wenn man nicht z. B. eine Regelung in die allgemeinen Vorschriften des BGB zum Behandlungsvertrag (§§ 630 a ff. BGB) integriert oder, wie *Kersten/Rixen* vorschlagen (unten IV. 5.), eine Lösung über das AGG versucht

3. Allenfalls sehr langfristig wirken würden dagegen „spezifische[…] Vorgaben für die Aus- und Weiterbildung in der Medizin und Pflege und insbesondere des intensivmedizinischen Personals, um auf die Vermeidung von Benachteiligungen wegen Behinderung in einer Triage-Situation hinzuwirken" (Rdnr. 128). Ob eine daraus resultierende, weit in die Zukunft reichende vage Aussicht auf Besserung genügt, wo der Gesetzgeber doch gehalten ist, „seiner Handlungspflicht *unverzüglich* durch geeignete Vorkehrungen nachzukommen" (Rdnr. 130[31]), bleibt zweifelhaft. Zwar befolgt ein „unverzüglicher" Erlass eines Gesetzes formal den Handlungsbefehl des Gerichts. Dem Sinn und Zweck, alsbald im Interesse behinderter Menschen Abhilfe zu schaffen, genügt ein erst in ferner Zukunft Auswirkungen entfaltendes Gesetz aber sicher nicht. Hinzu kommt, dass auch die genannten Vorgaben als Regelungen der ärztlichen Berufsausübung in die Kompetenz der Bundesländer fallen, so dass nicht einmal der schnelle Erlass von (einheitlichen?) Gesetzen zu erwarten ist. Erstaunlich ist schließlich, dass die *Fortbildung* vom Gericht nicht erwähnt wird – könnte sie doch bezogen auf Personal, das bereits in der Intensivmedizin tätig ist, eine viel zügigere Abhilfe schaffen.

4. Vielleicht ist aber auch der implizite Vorwurf des Gerichts, der Gesetzgeber habe „bislang [überhaupt] keine Vorkehrungen getroffen, die dem Risiko einer Benachteiligung von Menschen aufgrund einer Behinderung bei der Verteilung von knappen intensivmedizinischen Behandlungsressourcen wirksam begegnen" (Rdnrn. 108, 122), dahin zu verstehen, dass schon der nicht ganz untaugliche Versuch, gegenzusteuern, ausreichen wird. Denn das Gericht betont auch: „Besteht [wie hier] eine konkrete Schutzpflicht, kann das BVerfG deren Verletzung nur feststellen, wenn Schutzvorkehrungen entweder überhaupt nicht getroffen sind, wenn die getroffenen Regelungen und Maßnahmen offensichtlich ungeeignet oder völlig unzulänglich sind, das gebotene Schutzziel zu erreichen, oder wenn sie erheblich hinter dem Schutzziel zurückbleiben" (Rdnr. 98[32]). Das und der mehrfach betonte „weite" (Rdnr. 99) „Einschätzungs-, Wertungs- und Gestaltungsspielraum" des Gesetzgebers (Rdnrn. 99, 108, 126, 127)[33] werden die praktischen Folgen des Urteils möglicherweise minimieren – und besorgte Menschen mit Behinderung dann sicher nicht zufrieden stellen.

5. Eine letzte, jedenfalls politische Frage ergibt sich daraus, dass zahlreiche Passagen des Urteils darauf hindeuten, dass die Entscheidung (gemäß dem Antrag der Beschwerdeführer) nur eine mit einer *Pandemie* einhergehende Knappheit[34] *intensivmedizinischer* Ressourcen sowie eine *daraus* resultierenden Triage-Situation erfasst:

„Nach der Verfassungsbeschwerde ist hier allein zu entscheiden, ob der Gesetzgeber verpflichtet ist, wirksame Vorkehrungen zu treffen, dass niemand bei einer Entscheidung über die Verteilung von *pandemiebedingt* knappen *intensivmedizinischen* Behandlungsressourcen, also in einem Fall einer *Triage*, aufgrund einer Behinderung benachteiligt wird" (Rdnr. 87)[35]. – „Besteht das Risiko, dass Menschen in einer *Triage-Situation* bei der Zuteilung *intensivmedizinischer* Behandlungsressourcen wegen einer Behinderung benachteiligt werden, verdichtet sich der Schutzauftrag aus Art. 3 Abs. 3 S. 2 GG zu einer konkreten Pflicht des Staates, *hiergegen* wirksame Vorkehrungen zu treffen" (Rdnr. 109). – „Es liegen auch Anhaltspunkte dafür vor, dass für die Beschwerdeführenden ein Risiko besteht, bei Entscheidungen über die Verteilung *pandemiebedingt* nicht ausreichender überlebenswichtiger Ressourcen in der *Intensivmedizin* und damit bei einer Entscheidung über Leben und Tod aufgrund ihrer Behinderung benachteiligt zu werden" (Rdnr. 110). – „Das Risiko behinderter Menschen, bei der Zuteilung *pandemiebedingt* knapper *intensivmedizinischer* Ressourcen benachteiligt zu werden, ergibt sich nach den Feststellungen des Senats aus einer Gesamtschau verschiedener […] Umstände […] Dem Gesetzgeber stehen daher mehrere Möglichkeiten offen, *diesem Risiko* wirkungsvoll zu begegnen" (Rdnr. 126). – „Der Gesetzgeber muss […] dafür Sorge tragen, dass jede Benachteiligung wegen einer Behinderung bei der Verteilung *pandemiebedingt* knapper *intensivmedizinischer* Behandlungsressourcen hinreichend wirksam verhindert wird" (Rdnr. 130).

29) Kritik an den vom Ethikrat geprägten Bezeichnungen bei *Sowada*, NStZ 2020, 452.
30) So denn auch der konkrete Gesetzesvorschlag von *Kersten/Rixen* s. unten IV. 5.
31) Hervorhebung vom Verfasser.
32) Ebenso Rdnr. 71 der Entscheidung.
33) Im Verfahren über den Eilantrag auf verbindliche Regelung der Triage im Rahmen der Covid-19-Pandemie (NVwZ 2020, 1353) hatte das Gericht noch ausgeführt, dass die Verfassungsbeschwerde u. a. die Frage aufwerfe, „wie weit der Einschätzungs-, Wertungs- und Gestaltungsspielraum des Gesetzgebers für die Regelung konkreter medizinischer Priorisierungsentscheidungen reicht" (Hervorhebung vom Verfasser). Dies bedürfe „einer eingehenden Prüfung, die im Rahmen eines Eilverfahrens nicht möglich ist." Angesichts dessen überraschen die mehr als rudimentären Ausführungen in der Hauptsache doch sehr.
34) Der Antrag (oben Fn. 2) war sogar konkret bezogen auf die „durch das COVID19 ausgelöste[…] Pandemie".
35) Hervorhebungen (auch im Folgenden) vom Verfasser.

Die Frage nach der Reichweite der Entscheidung stellt sich insbesondere deshalb, weil Knappheitssituationen und daraus folgende diskriminierungsanfällige überlebenswichtige Zuteilungsentscheidungen im Gesundheitssystem nicht nur auf Intensivstationen und nicht nur in Pandemiezeiten vorkommen. Intensivmedizinische Ressourcen können auch in „Normalzeiten" knapp sein, insbesondere aufgrund des Kostendrucks, dem die Krankenhäuser unterliegen. Was gilt also, wenn außerhalb der gegenwärtigen Pandemie oder unabhängig von ihr die Intensivstation ausschließlich mit Herzinfarkt-Patienten belegt ist und ein weiterer Herzinfarkt-Patient hinzukommt? Müsste der Gesetzgeber nicht auch hier vor Benachteiligung wegen einer Behinderung schützen? Zudem wird auch im Rettungsdienst bei einem größeren Schadensereignis „triagiert", wenn mehr Opfer behandlungsbedürftig sind, als zunächst behandelt werden können[36]. Auch wird in den Notaufnahmen der Krankenhäuser häufig auf das „Manchester Triage System", ein standardisiertes Verfahren zur systematischen Ersteinschätzung bzw. Triage der Behandlungsdringlichkeit von Patienten in Rettungsstellen bzw. Notaufnahmen, zurückgegriffen[37]. Müsste der Gesetzgeber die geforderte Regelung nicht auch auf all diese Bereiche einschließlich des Katastrophenschutzes erstrecken? Oder darf er sich mit einem „Gesetz zur Verhinderung von Benachteiligung aufgrund einer Behinderung bei der Verteilung pandemiebedingt knapper intensivmedizinischer Ressourcen" begnügen? Der in der Literatur von *Kersten/Rixen* vorgeschlagene Gesetzeswortlaut, den die Autoren als „minimalinvasive" bundesrechtliche Regelung zur Einfügung in das AGG vorschlagen, geht jedenfalls erheblich weiter[38]: „In einer Situation, in der knappe, der Lebenserhaltung dienende medizinische Behandlungsmittel zwingend nur einer Person zugeteilt werden können, hat der oder die Behandlungsverantwortliche eine Entscheidung zu treffen, die sich an der klinischen Erfolgsaussicht orientiert und Benachteiligungen im Sinne eines in § 1 dieses Gesetzes [= des AGG] genannten Grundes vermeidet; die Entscheidung kann auch darin bestehen, die Behandlung eines Patienten oder einer Patientin zugunsten eines anderen Patienten oder einer anderen Patientin nicht einzuleiten oder abzubrechen."

### V. Fazit

Die Entscheidung des BVerfG v. 16. 12. 2021 beschränkt sich in ihren Aussagen strikt auf die gestellten Anträge der Beschwerdeführer. Das ist juristisch nicht zu missbilligen, gibt aber der weiteren Rechtsentwicklung in wichtigen Fragen wenig Hilfestellung. Zu kritisieren ist, dass das Gericht zentrale verfassungsrechtliche Aspekte, die zum Kern der von den Beschwerdeführern aufgeworfenen Fragen gehören, geradezu aufreizend lässig behandelt.

**Open Access.** Dieser Artikel wird unter der Creative Commons Namensnennung 4.0 International Lizenz veröffentlicht, welche die Nutzung, Vervielfältigung, Bearbeitung, Verbreitung und Wiedergabe in jeglichem Medium und Format erlaubt, sofern Sie den/die ursprünglichen Autor(en) und die Quelle ordnungsgemäß nennen, einen Link zur Creative Commons Lizenz beifügen und angeben, ob Änderungen vorgenommen wurden.
Die in diesem Artikel enthaltenen Bilder und sonstiges Drittmaterial unterliegen ebenfalls der genannten Creative Commons Lizenz, sofern sich aus der Abbildungslegende nichts anderes ergibt. Sofern das betreffende Material nicht unter der genannten Creative Commons Lizenz steht und die betreffende Handlung nicht nach gesetzlichen Vorschriften erlaubt ist, ist für die oben aufgeführten Weiterverwendungen des Materials die Einwilligung des jeweiligen Rechteinhabers einzuholen. Weitere Details zur Lizenz entnehmen Sie bitte der Lizenzinformation auf http://creativecommons.org/licenses/by/4.0/deed.de.

36) Dazu und zu weiteren Fragen bezüglich der Reichweite des Urteils *Tackenberg*, https://www.anwalt.de/rechtstipps/bverfg-zur-triage-eine-einschaetzung-195954.html, Zugriff am 7. 1. 2022.
37) S. statt vieler die Homepage der Charité, https://notfallmedizin-nord.charite.de/notfallversorgung/manchester_triage_system/, Zugriff am 7. 1. 2022. – Da das System zum Teil nicht differenziert genug ist, wechseln manche Kliniken auf andere Systeme der Kategorisierung, etwa den Emergency Severity Index (ESI), s. *Dierbach*, https://www.pflegemagazin-rlp.de/wie-effektiv-ist-das-manchester-triage-system, Zugriff am 7. 1. 2022.
38) *Kersten/Rixen*, Der Verfassungsstaat in der Corona-Krise, 2. Aufl. 2021, VI. 2. Triage.

## Anregungen für ein allgemeines Heilberufegesetz

Sektorale Heilkundeerlaubnisse und Heilpraktikerberuf

**Christof Stock**

### I. Einführung

Die Koalition von SPD, Bündnis 90/Die Grünen und FDP will ein allgemeines Heilberufegesetz auf den Weg bringen[1]. Es könnte zur Abschaffung des HeilprG führen. Zwei dort geregelte Materien sind reformbedürftig: Der Begriff der Heilkunde ist zentral für die Ärzteschaft und die „anderen Heilberufe", die in Art. 74 Abs. 1 Nr. 19 GG erwähnt sind. Er ist in § 1 Abs. 2 HeilprG definiert[2]. Mit einer reformierten Legaldefinition oder neuen Berufsqualifikationen könnte die „systematische Unstimmigkeit"[3] behoben werden, die zwischen den Gesundheitsfachberufen und dem Heilpraktikerberuf besteht, der ebenfalls im HeilprG geregelt ist[4].

Heilpraktikerinnen und Heilpraktiker genießen die Kurierfreiheit, d. h. die Möglichkeit, die Heilkunde fast ohne

Prof. Dr. iur. Christof Stock,
Delheid Soiron Hammer Partnerschaft mbB,
Friedrichstrasse 17–19, 52070 Aachen, Deutschland

1) *SPD, Bündnis 90/Die Grünen, FDP,* Koalitionsvertrag 2021 „Mehr Fortschritt wagen", S. 82.
2) Vgl. § 2 Abs. 5 BÄO, § 1 Abs. 2 S. 1 PsychThG, § 2 Abs. 3 S. 1 ZHG.
3) BVerwGE 134, S. 345 = MedR 2010, 334–338; BVerwGE 166, 354–368 = MedR 2021, 61–66; Anm. *Liebler* jurisPR-BVerwG 13/2020, Anm. 3; BVerwG, Urt. v. 25. 2. 2021 – 3 C 17/19 –, für MedR Heft 4 mit Anm. des Verf.
4) Der Artikel fasst das Rechtsgutachten zum Heilpraktikerrecht zusammen, das der Autor im Auftrag des BMG im April 2021 erstellt hat. https://www.bundesgesundheitsministerium.de/service/publikationen/details/rechtsgutachten-zum-heilpraktikerrecht.html Die inzwischen ergangene Rechtsprechung ist eingearbeitet.

Funding

Open access funding enabled and organized by Projekt DEAL.

